# Pregnancy after breast cancer treatment in young patients

**DOI:** 10.3389/fonc.2025.1656429

**Published:** 2025-08-25

**Authors:** Young-Jin Lee, Tae-Kyung Yoo, Sae Byul Lee, Jisun Kim, Il-Yong Chung, Beom Seok Ko, Jong Won Lee, Byung Ho Son, Seonok Kim, Hee Jeong Kim

**Affiliations:** ^1^ Department of Surgery, Asan Medical Center, University of Ulsan, College of Medicine, Seoul, Republic of Korea; ^2^ Department of Clinical Epidemiology and Biostatistics, Asan Medical Center, Seoul, Republic of Korea

**Keywords:** breast cacner, pregnanacy, fertility preservation, endocrine therapy, young patient

## Abstract

**Introduction:**

Breast cancer (BC) treatments can impair fertility in young women, causing considerable distress and potentially influencing treatment decisions, yet comprehensive real-world data on pregnancy outcomes after BC remain limited. This study aims to provide comprehensive real-world data on pregnancy following BC treatment to guide clinical practice and patient counseling.

**Methods:**

We conducted a retrospective cohort study using medical records from a single tertiary medical center in South Korea. The study included 995 premenopausal women aged 18 to 40 years who were diagnosed with stage 0–III BC between December 2010 and September 2020. The primary outcomes included post-treatment pregnancy rates, factors associated with subsequent pregnancy, timing of conception, pregnancy outcomes, and oncologic outcomes among those who conceived.

**Results:**

The median age was 32 years (interquartile range [IQR], 30–34 years). Of 995 patients, 115 had at least one pregnancy after their BC diagnosis. Significant differences in pregnancy rates and the interval from BC treatment to pregnancy were observed according to hormone receptor status and pregnancy history prior to BC diagnosis. Among those who conceived, 46.1% discontinued endocrine therapy (ET) to achieve pregnancy. Following BC treatment, pregnancies were observed in 7.8% of women who were >35 years old at diagnosis, 17.8% of women who were unmarried at diagnosis, and 6.8% of women who already had children. Of the 76 patients who discontinued ET to attempt pregnancy, 53 (69.7%) successfully conceived. Among those who achieved pregnancy after ET discontinuation, four patients (7.5%) experienced cancer recurrence.

**Discussion:**

Effective fertility preservation counseling is necessary for patients of reproductive age with BC, regardless of age, marital status, or whether they had children before BC diagnosis. This study can be referenced to appropriately address and manage the impact of chemotherapy and ET on pregnancy after BC treatment.

## Introduction

Breast cancer (BC) remains the most prevalent malignancy among women of reproductive age globally, with Asian regions demonstrating particularly high proportions of young-onset cases (1, 2). While improvements in early detection and treatment have increased survival rates, these advances have raised fertility considerations among young survivors (3). BC treatments, particularly endocrine therapy and chemotherapy, are known to potentially impair fertility, causing considerable distress among these young survivors (4). Approximately 63% of young women of childbearing age diagnosed with BC have concerns about their fertility post-treatment, and 39% report that these concerns influence their treatment decision-making (5–7). Notably, 2% of patients may decline recommended chemotherapy or endocrine therapy (ET) owing to fertility concerns, potentially compromising their oncologic outcomes (7). BC survivors exhibit significantly lower pregnancy rates (RR, 0.40; 95% confidence interval [CI], 0.32–0.49) compared to the general population, substantiating these fertility concerns (8). Prior studies have demonstrated the safety of pregnancy and fertility preservation in BC survivors (9, 10). A recent prospective study, the POSITIVE trial, demonstrated the safety of interrupting hormonal therapy to achieve pregnancy (11), as well as the safety of using assisted reproductive techniques in BC survivors (12). Nevertheless, concerns persist regarding potential treatment delays, increased recurrence rates, and decreased treatment adherence (4, 13). Moreover, while fertility preservation strategies exist, there remains uncertainty regarding their utilization and efficacy in patients with BC (14).

This study aimed to provide comprehensive real-world data on pregnancy following BC treatment, with the goal of informing clinical practice and patient counseling regarding this critical aspect of survivorship.

## Materials and methods

### Study population

**Figure 1:**
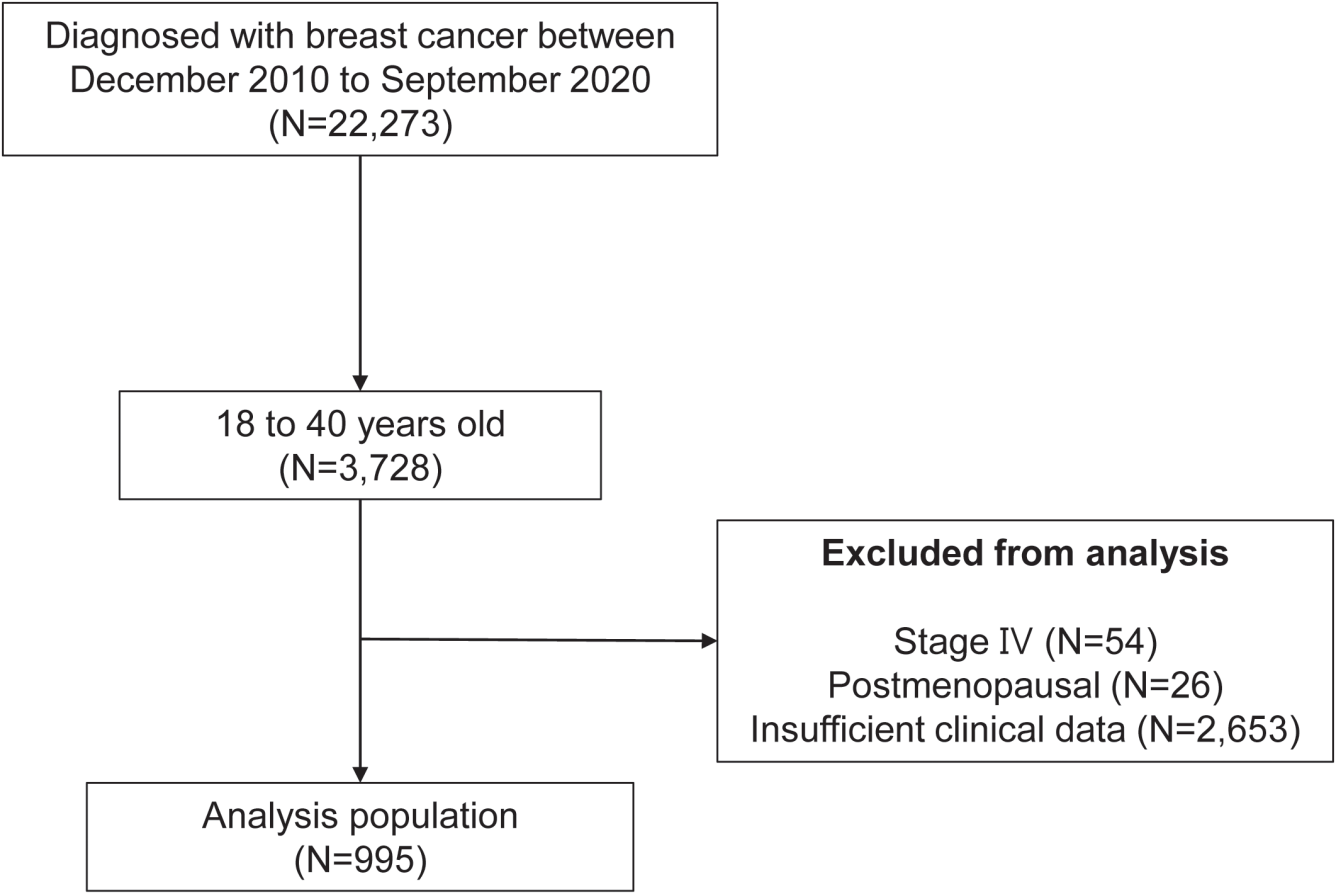
Study population.

In this retrospective study, we examined the medical records of patients diagnosed with BC at a single tertiary medical center between December 2010 and September 2020 (**Figure 1**). The inclusion criteria were as follows: female BC patients aged between 18 and 40 years who were diagnosed with BC during the study period. A total of 3,728 patients met these criteria from an initial cohort of 22,273 female BC patients. We then applied exclusion criteria to ensure the reliability and validity of the study. First, we excluded patients with stage IV BC owing to the distinct clinical course and prognosis associated with this advanced disease stage. Next, postmenopausal women were excluded, as the study was focused on fertility concerns relevant to premenopausal women. Lastly, patients with insufficient clinical data were also excluded to ensure comprehensive and accurate analysis. After applying these exclusion criteria, the final analysis population consisted of 995 patients. These patients formed the basis of the study and all subsequent analysis.

### Variables and definitions

The treatment administered to patients was in accordance with national guidelines. Patient and disease characteristics were retrieved from digital medical records. During their initial consultation after referral to Asan Medical Center, patients’ marital status (married, single, or divorced) and reproductive history (number of children, year of birth) were documented. Information on pregnancies following breast cancer treatment was obtained from standardized medical record forms for young patients, which physicians are required to complete during follow-up outpatient visits conducted at six-month intervals. These forms include data on pregnancy status and the expected or actual date of delivery, and were used for analysis in this study.

### Statistical analysis

Data analysis was performed using R version 3.5.1 (R Foundation for Statistical Computing, Vienna, Austria; http://www.r-project.org). Pregnancy curves were generated using the Kaplan–Meier method, and the significance of pregnancy rate differences among selected variables was verified using the log-rank test. Hazard ratios were estimated using univariate Cox regression analysis. Further, multivariate Cox regression analysis, implemented with a backward elimination approach, was used to estimate both hazard ratios and p-values, aiding in the identification of independent prognostic indicators. Any unknown groups within each variable were excluded before initiating the Cox analysis. All reported p-values are two-sided, with statistical significance attributed to p-values < 0.05.

### Ethics approval

All actions performed in the research involving human participants complied with the ethical guidelines of the institutional and national research committees. In addition, the study adhered to the principles of the Helsinki Declaration of 1964, along with its subsequent updates, or met similar ethical criteria.

The study was reviewed and approved by the Institutional Review Board of Asan Medical Center (approval number 2021-1382). The authors had access to anonymized participant data. Informed consent was waived as the study used retrospective clinical data.

## Results

### Patient characteristics of the overall cohort and pregnancy group

The overall cohort included 995 young women diagnosed with BC, of which 115 experienced a pregnancy after treatment during the median follow-up period of 63 months (**Table 1**). The mean age at diagnosis for the overall cohort was 32 years (interquartile range [IQR], 30–34 years), whereas for those who experienced a pregnancy, the mean age was 31 years (IQR, 29.5–33 years). Among patients who became pregnant after BC treatment, 47.0% were unmarried, and 18.3% had children at the time of diagnosis. Concerning the TNM stage distribution in the pregnancy group, 10.4% of the cases were stage III. Hormone receptor status was positive in 66.5% of the overall cohort and 56.5% of the pregnancy group. Chemotherapy was received by 61.7% of the pregnancy group. ET was administered to 55.7% of the pregnancy group. Fertility counseling was provided to 51.2% of the overall cohort and 55.7% of the pregnancy group. Oocyte/embryo cryopreservation as a fertility preservation procedure was utilized by 8.1% of the overall cohort and 7.0% of the pregnancy group. The implementation of educational material was associated with higher fertility preservation rates compared to pre-implementation (65.5% vs. 34.5%, respectively) (**Supplementary Figure 1**).

**Table 1:** Patient characteristics of the overall cohort and pregnancy group.

Variables	No. (%)	
	Overall cohort (n=995)	Pregnancy group (n=115)
Age at diagnosis, year (median, IQR)	32 (30–34)	31 (29.5–33)
Age at diagnosis, years		
<30	231 (23.2)	29 (25.2)
30–34	602 (60.5)	75 (65.2)
≥35	162 (16.3)	11 (9.6)
Pregnancy		
No	880 (88.4)	–
Yes	115 (11.6)	–
Marriage at diagnosis		
No	341 (34.3)	54 (47.0)
Yes	413 (41.5)	51 (44.3)
Unknown	241 (24.2)	10 (8.7)
History of pregnancy at diagnosis		
No	317 (31.9)	50 (43.5)
Yes	324 (32.5)	21 (18.3)
Unknown	354 (35.6)	44 (38.3)
Clinical or Pathologic TNM stage		
Stage 0	93 (9.4)	17 (14.8)
Stage I	283 (28.4)	34 (29.6)
Stage II	466 (46.8)	52 (45.2)
Stage III	153 (15.4)	12 (10.4)
Breast surgery		
BCS	620 (62.3)	82 (71.3)
Mastectomy	375 (37.7)	33 (28.7)
Hormone receptor		
Negative	333 (33.5)	50 (43.5)
Positive	662 (66.5)	65 (56.5)
HER2 overexpression		
Negative	814 (81.8)	99 (86.1)
Positive	181 (18.2)	16 (13.9)
Subtype by IHC		
Hormone receptor +/HER2–	540 (54.3)	56 (48.7)
Hormone receptor+/HER2+	122 (12.3)	9 (7.8)
Hormone receptor–/HER2+	59 (5.9)	7 (6.1)
Hormone receptor–/HER2–	274 (27.5)	43 (37.4)
Radiation therapy		
No	269 (27.0)	27 (23.5)
Yes	714 (71.8)	87 (75.7)
Unknown	12 (1.2)	1 (0.9)
Chemotherapy		
No	310 (31.2)	44 (38.3)
Yes	679 (68.2)	71 (61.7)
Unknown	6 (0.6)	0
Endocrine therapy		
No	337 (33.9)	51 (44.3)
Yes	655 (65.8)	64 (55.7)
Unknown	3 (0.3)	0
Fertility counseling		
No	486 (48.8)	51 (44.3)
Yes	509 (51.2)	64 (55.7)
GnRHa during chemotherapy		
No	535 (53.8)	61 (53.0)
Yes	447 (44.9)	52 (45.2)
Unknown	13 (1.3)	2 (1.7)
Fertility preservation		
No	914 (91.9)	107 (93.0)
Oocyte cryopreservation	44 (4.4)	1 (0.9)
Embryo cryopreservation	37 (3.7)	7 (6.1)

### Factors associated with pregnancy

**Figure 2:**
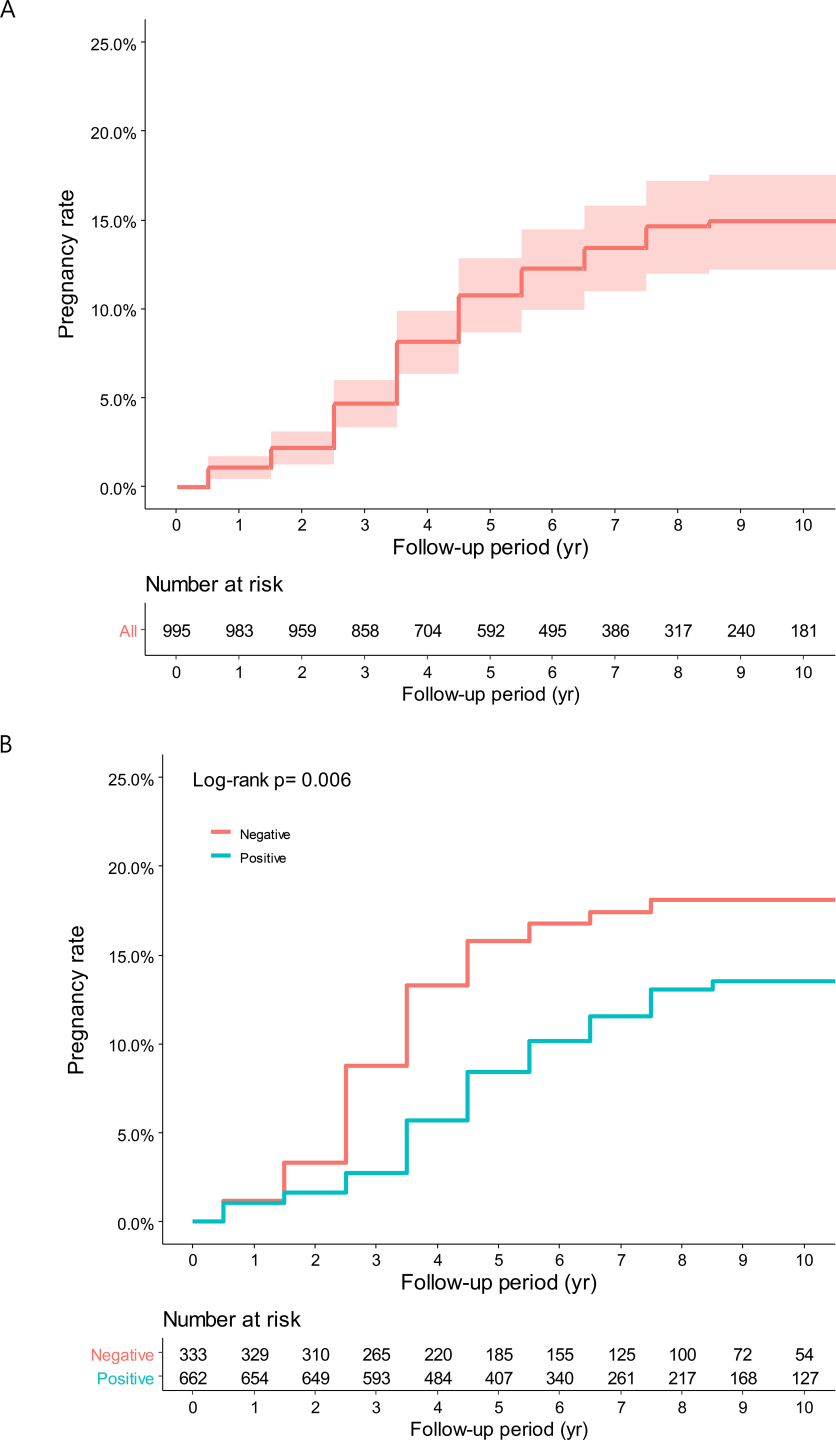
**(A)** Cumulative incidence of overall pregnancy. **(B)** Cumulative incidence of pregnancy according to hormone receptor status.

Age at diagnosis, marital status, previous pregnancies, TNM stage, hormone receptor status, and chemotherapy were evaluated for their association with pregnancy rates and interval after BC treatment (**Table 2**). Overall, the cumulative incidence of pregnancy at 10 years was 14.2%, with a median time from BC to pregnancy of 3 years (IQR, 2–4 years) (**Figure 2A**). In patients with hormone receptor-positive and negative BC, the cumulative incidence of pregnancy at 10 years was 13.5% (65 out of 662 hormone receptor-positive patients) and 18.1% (50 out of 333 hormone receptor-negative patients), respectively (HR = 0.60, 95% CI: 0.41–0.87, P = 0.006) (**Figure 2B**). The median time from BC to pregnancy was significantly longer in patients with hormone receptor-positive (3 years, IQR 2–5 years) than patients with hormone receptor-negative (2 years, IQR 2–3 years) status.

**Table 2:** Results of uni- and multi-variable cox proportional hazards model evaluating time to pregnancy.

Variables		Cases	Events	Rate*	Univariable analysis				Multivariable analysis			
					HR	95% CI		*P*	HR	95% CI		P
		(N)	(N)	(%)		Lower	Upper			Lower	Upper	
Age at diagnosis, years	≤35	833	104	15.4	Ref				Ref			
	>35	162	11	7.8	0.56	0.30	1.03	0.063	0.69	0.37	1.30	0.251
Marriage at diagnosis	No	341	54	17.8	Ref							
	Yes	413	51	13.3	0.76	0.52	1.11	0.160				
Pregnancy history at diagnosis	No	317	50	17.4	Ref				Ref			
	Yes	324	21	6.8	0.37	0.23	0.62	<0.001	0.39	0.24	0.67	<0.001
TNM stage	0	93	17	20.7	Ref				Ref			
	I–III	902	98	14.3	0.59	0.35	0.99	0.045	0.90	0.49	1.64	0.720
Hormone receptor status	Negative	333	50	18.1	Ref				Ref			
	Positive	662	65	13.5	0.60	0.41	0.87	0.006	0.52	0.36	0.77	0.001
Chemotherapy	No	310	44	18.2	Ref				Ref			
	Yes	679	71	13.8	0.69	0.47	1.01	0.054	0.71	0.45	1.12	0.140

*The pregnancy rate was calculated using Kaplan–Meier (KM) estimates.

The pregnancy history before diagnosis proved to be significant in pregnancy after treatment. For those with no history of pregnancy, cumulative incidence of pregnancy at 10 years was 17.4% (50 out of 317 patients), compared to 6.8% (21 out of 324 patients) (HR = 0.37, 95% CI: 0.23–0.62, P < 0.001). The TNM stage at diagnosis showed a similar trend. Among stage 0 patients, the cumulative incidence of pregnancy at 10 years was 20.7% (17 out of 93 patients), compared to 14.3% (98 out of 902 patients) with stages I–III (HR = 0.59, 95% CI: 0.35–0.99, P = 0.045). Age above 35 years and chemotherapy showed a trend toward lower pregnancy rates.

Multi-variable analysis was carried out to adjust for potential confounders. The hormone receptor-positive status continued to be associated with lower pregnancy rates (HR = 0.52, 95% CI: 0.36–0.77, P = 0.001). A history of pregnancy remained a significant predictor, with patients who had been pregnant before being less likely to become pregnant after treatment (HR = 0.39, 95% CI: 0.24–0.67, P < 0.001).

### Outcomes in pregnancy after BC treatment

The outcomes for the 115 patients who experienced a pregnancy after treatment are detailed in **Table 3**. The interval between surgery and conception varied, with 9.6% conceiving within 1 year after surgery, and the highest number (25.2%) of pregnancies occurred between 3 and 4 years post-surgery. Among first pregnancies after BC treatment, 7.8% resulted in no live births, 66.1% in one live birth, 15.7% in two live births, and 0.9% in three live births. The oncologic outcomes showed that 85.2% of the patients who became pregnant were without evidence of disease (NED). Local recurrence occurred in 7.8% of the patients, regional recurrence in 4.3%, and distant metastasis in 1.7%. There was one case (0.9%) of secondary malignancy, and no patients had died at the time of the study.

**Table 3:** Outcomes in patients with a pregnancy (N=115).

Outcomes	No. (%)
Pregnancy (conception) interval	
<1 Y after surgery	11 (9.6)
1–2 Y after surgery	11 (9.6)
2–3 Y after surgery	24 (20.9)
3–4 Y after surgery	29 (25.2)
4–5 Y after surgery	19 (16.5)
5–6 Y after surgery	9 (7.8)
6–7 Y after surgery	6 (5.2)
7–8 Y after surgery	5 (4.3)
8–9 Y after surgery	1 (0.9)
No. of live births from first pregnancy after BC treatment	
0	9 (7.8)
1	76 (66.1)
2	18 (15.7)
3	1 (0.9)
Unknown	11 (9.6)
Oncologic outcomes	
NED	98 (85.2)
Local recurrence	9 (7.8)
Regional recurrence	5 (4.3)
Distant metastasis	2 (1.7)
Secondary malignancy	1 (0.9)
Dead	0 (0.0)
Endocrine therapy (ETx)	N=64
Discontinuation of ETx for pregnancy	53 (46.1)
Finished planned regimen	10 (8.7)
Stop for other reasons	1 (0.9)
Duration of ETx before stop for pregnancy	N=53
<1 Y	5 (9.4)
1–2 Y	17 (32.1)
2–3 Y	20 (37.7)
3–4 Y	8 (15.1)
4–5 Y	3 (5.7)
≥5Y	0 (0.0)

ET was discontinued for pregnancy in 46.1% of the patients, whereas 8.7% finished their planned regimen and 0.9% stopped for other reasons. Among those who discontinued ET for pregnancy, 9.4% stopped within the first year, 32.1% after 1–2 years, 37.7% after 2–3 years, 15.1% after 3–4 years, and 5.7% after 4–5 years. No patients continued ET for ≥5 years. We analyzed the relationship between the duration of ET and subsequent pregnancy rates upon its discontinuation because of pregnancy (**Supplementary Table 2**). Patients who received treatment for a duration of 1–2 years demonstrated the highest pregnancy rate of 85.0% (17 out of 20). In the overall cohort, 76 patients discontinued ET to attempt pregnancy, and among them, 17% experienced a recurrence of BC (**Supplementary Figure 2**).

## Discussion

This large-scale retrospective study provides real-world data on pregnancy outcomes among young BC survivors. Patients with hormone receptor-positive BC had significantly lower pregnancy rates and longer pregnancy intervals after treatment. Outcomes for the 115 patients who conceived post-BC treatment showed varied intervals between surgery and conception, with the highest number of pregnancies occurring 3–4 years post-surgery. During the follow-up period, 82.7% of the conceptions resulted in one or more live births, and 85.2% were without evidence of disease, with no patient deaths reported. Among the patients who became pregnant, 46.1% had discontinued hormone therapy for pregnancy.

An interesting finding from this study is that even among patients who were >35 years old, unmarried at diagnosis, or had children, 7.8%, 17.8%, and 6.8%, respectively, became pregnant after BC treatment. These figures are noteworthy when compared to the overall cohort’s pregnancy rate of 14.2%. Therefore, adequate counseling and planning for having children should be conducted before starting treatment, even for BC patients who are older, unmarried, or have not previously given birth. In this analysis, a history of pregnancy before BC diagnosis significantly reduced the chances of conception after BC treatment. This might be attributed to the fact that women with one or more children are less motivated to have a baby after overcoming BC. Additionally, the vague fear among patients and physicians that pregnancy could negatively impact the prognosis of BC could contribute to low motivation. Survey studies conducted among BC specialists revealed that 20–30% of physicians incorrectly held the belief that pregnancy adversely affects BC prognosis (15, 16). However, a prior survey study on BC survivors revealed that 56% of young women who had one or more children before their diagnosis wished to have more children after their BC treatment (5). Our analysis revealed that subsequent pregnancies occurred even among patients who already had children. Among patients with one child, 11% (n=18/163) achieved an additional pregnancy, and notably, 1.9% (n=3/161) of patients with two or more children conceived after BC treatment (**Supplementary Table 1**). These findings suggest that pre-existing parenthood status should not be a limiting factor when considering fertility preservation options for patients, and fertility preservation counseling should be offered to all patients regardless of their parental status.

Another significant factor influencing pregnancy outcomes after BC treatment was hormone receptor status. Our findings suggested that hormone receptor-positive patients were less likely to conceive post-treatment compared to their hormone receptor-negative counterparts. This could be attributed, first, to concerns shared by patients and physicians that increased estrogen levels during pregnancy may have negative effects on the prognosis of hormone receptor-positive BC (17). However, previous studies have shown that pregnancy and breastfeeding do not have a negative impact on the prognosis of hormone receptor-positive BC (18). Second, the fear of discontinuing treatment owing to pregnancy as a contraindication during hormone therapy could lead to the decision to forego pregnancy (19). Our analysis revealed that more than half of the patients received fertility preservation counseling prior to treatment, indicating their desire for future pregnancies. However, among patients receiving ET, only 7.6% discontinued treatment because of pregnancy. This suggests a tendency among younger patients to give up having a baby and opt for treatment continuation. In the POSITIVE trial, women with hormone receptor-positive early BC who temporarily stopped ET because of pregnancy had an 8.9% incidence of BC events (including distant recurrence) over 3 years, which did not show an increased risk compared to the external control group (11). Similarly, in the retrospective analysis of this study, the cumulative incidence of BC events over 10 years was 10.1%. These findings, from both the POSITIVE trial and our study, may provide reassuring evidence for patients who consider discontinuing ET to pursue pregnancy. Additionally, the results of this study revealed that the majority of pregnant patients discontinued their ET after 1–3 years (**Supplementary Table 2**). Previously, there was no consensus on the duration of ET maintenance before attempting pregnancy. However, it is considered safe to interrupt ET after maintaining it for a minimum of one and a half years, based on the findings of the POSITIVE trial (11). Among participants in the POSITIVE trial who discontinued ET to pursue pregnancy, 74% achieved at least one successful conception, with the highest success rates documented in patients who underwent embryo/oocyte cryopreservation before initiating treatment (12). Therefore, comprehensive fertility and pregnancy counseling should be integrated into the initial BC management plan at the time of diagnosis.

In the results of this study, chemotherapy did not show a significant impact on pregnancy rates. This finding differs from the conventional belief that chemotherapy compromises ovarian function (20–22). GnRHa can be used concurrently with chemotherapy for ovarian protection (23). In our study, GnRHa was used for fertility preservation in 447 out of 679 patients (65.8%). The PROMISE-GIM6 study conducted by Lambertini et al. demonstrated a higher frequency of menstrual resumption in the GnRHa group (24), and the 2015 POEMS study by Moore et al. reported that GnRHa use, compared to chemotherapy alone, prevented ovarian failure, reduced premature ovarian failure, and showed higher pregnancy rates (25). However, as the primary outcome in most clinical trials using GnRHa was menstrual resumption rather than fertility, evidence verification is needed regarding its use for the purpose of fertility preservation.

Oocyte or embryo cryopreservation represents an established method of fertility preservation that can be implemented during chemotherapy treatment (26–28). In a recent international multicenter study (29) conducted on young patients with BC who are BRCA carriers, 659 out of 4,732 BRCA carriers had at least one pregnancy; among these, 8.2% became pregnant using previously cryopreserved oocytes or embryos after BC treatment. In contrast, our study found this rate to be slightly lower at 7%. The lower rate of fertility preservation procedures in this study, compared to that of international BRCA carriers who are at a higher risk for hereditary BC, may indicate that adequate counseling on the effectiveness and side effects of oocyte and embryo cryopreservation is not being provided. Moreover, a secondary analysis of the recently published POSITIVE trial demonstrated that embryo/oocyte cryopreservation followed by embryo transfer is a safe and effective method for achieving pregnancy after BC treatment (12). Furthermore, in contrast to the United States and European countries, South Korea imposes legal restrictions on pre-implantation or prenatal genetic testing (excluding BRCA mutations) (30–32), which may induce hesitancy in women diagnosed with cancer concerning their pregnancy and fertility preservation options. Therefore, the establishment of a comprehensive fertility preservation system that facilitates the dissemination of accurate information and supports informed decision-making is essential.

Fertility preservation counseling is also necessary to improve medication adherence for appropriate breast cancer treatment. Despite the beneficial impact on survival associated with tamoxifen use, a recent study revealed that 13.4% of women choose not to start taking tamoxifen, and an additional 15.5% discontinue its use before completing the recommended duration of 5 years (33). In this study, fertility emerged as a significant factor influencing early discontinuation of tamoxifen among young patients with BC. Alongside side effects, it is considered one of the most crucial causes for not initiating or discontinuing ET. According to another research finding, individuals who used tamoxifen did not exhibit a decrease in ovarian reserve compared to those who did not use tamoxifen (34). Therefore, providing education on these pieces of evidence to patients in the reproductive age group is necessary.

According to several survey studies conducted among healthcare providers treating breast cancer, there are significant barriers to implementing fertility preservation. These barriers include lack of knowledge (35) and insufficient system resources, such as time and human resources (36, 37). Consequently, there is a growing need for comprehensive multidisciplinary counseling programs that address both fertility preservation and BC treatment (38). Various research initiatives are currently underway to address these challenges. The PREFER study in Italy is collecting prospective data, including outcomes from patients who have successfully conceived, to improve oncofertility counseling (39). The MYBC trial in Korea is working to establish evidence for an effective multidisciplinary shared decision-making program for fertility preservation (40). The results from these trials, combined with real-world data, are expected to contribute to improving both treatment approaches and fertility preservation options for young patients with BC.

This study has some limitations. First, as a retrospective single-center study, it inherently carries the limitations associated with this study design, including potential selection bias and the inability to control for all confounding variables. Because randomized trials on pregnancy-related topics may raise ethical concerns, we are currently collecting prospective pregnancy data through an ongoing trial focusing on multidisciplinary decision-making in young breast cancer patients (40), and we plan to conduct future research based on these data. Second, decisions and outcomes related to pregnancy are significantly influenced by national policies, cultural factors, and societal norms, which may limit the generalizability of our findings to other populations and healthcare settings. Despite these limitations, our study has a notable strength. It represents one of the largest long-term follow-up studies conducted in a conservative Asian setting, providing valuable insights into pregnancy outcomes and fertility preservation in patients with BC within this cultural context. This unique perspective contributes significantly to the existing body of literature on fertility and pregnancy after BC treatment.

In conclusion, our study highlights that effective fertility preservation counseling is necessary for all patients with BC of reproductive age, regardless of age, marital status, or whether they had children before BC diagnosis. It provides real-world data that can be referenced to appropriately address and manage the impact of chemotherapy and ET on pregnancy after BC treatment. These findings can be valuable knowledge for clinical decision-making and counseling.

## Data Availability

The data analyzed in this study is subject to the following licenses/restrictions: For external transfer of the dataset, approval from the institutional IRB is required. Requests to access these datasets should be directed to Young-Jin Lee, imarctia@naver.com.
