# Supplementary material for: Pregnancy after breast cancer treatment in young patients
Source: Front Oncol. 2025 Aug 25;15:1656429. doi: 10.3389/fonc.2025.1656429 (PMC12414922; doi:10.3389/fonc.2025.1656429)
Supplement: Supplementary file 1 [file DataSheet1.docx]

**Supplemental Material**

**eTable 1. Pregnancy rate according to number of children before breast cancer**

**eTable 2. Pregnancy rate according to duration of ET prior to discontinuation for pregnancy**

**eFigure 1. Comparison of fertility preservation rates before and after implementation of educational material**

**eFigure 2. Recurrence after discontinuation of endocrine therapy**

**eTable 1. Pregnancy rate according to number of children before breast cancer**

|  | **Total** | **Pregnancy** | **Rate** |
| --- | --- | --- | --- |
|  | **N=995** | **N=115** | **%** |
| **Number of children before BC** |  |  |  |
| 0 | 317 | 50 | 15.8 |
| 1 | 163 | 18 | 11.0 |
| 2 | 138 | 2 | 1.4 |
| 3 | 21 | 1 | 4.8 |
| 4 | 2 | 0 | 0.0 |
| Unknown | 354 | 44 | 12.4 |

*BC,* Breast cancer.

**eTable 2. Pregnancy rate according to duration of ET prior to discontinuation for pregnancy**

|  | **Discontinuation for pregnancy** | **Pregnancy** |
| --- | --- | --- |
|  | **N=76** | **N=53** |
| Duration of ET, years |  |  |
| <1 | 7 | 5 |
| 1 ~ 2 | 20 | 17 |
| 2 ~ 3 | 30 | 20 |
| 3 ~ 4 | 12 | 8 |
| 4 ~ 5 | 5 | 3 |
| ≥5 | 2 | 0 |
| Average duration of ET before stopping | 27.6 months | 25.7 months |
| Breast cancer recurrence | 5 patients | 4 patients |

*ET,* endocrine therapy

**eFigure 1. Comparison of fertility preservation rates before and after implementation of educational material**


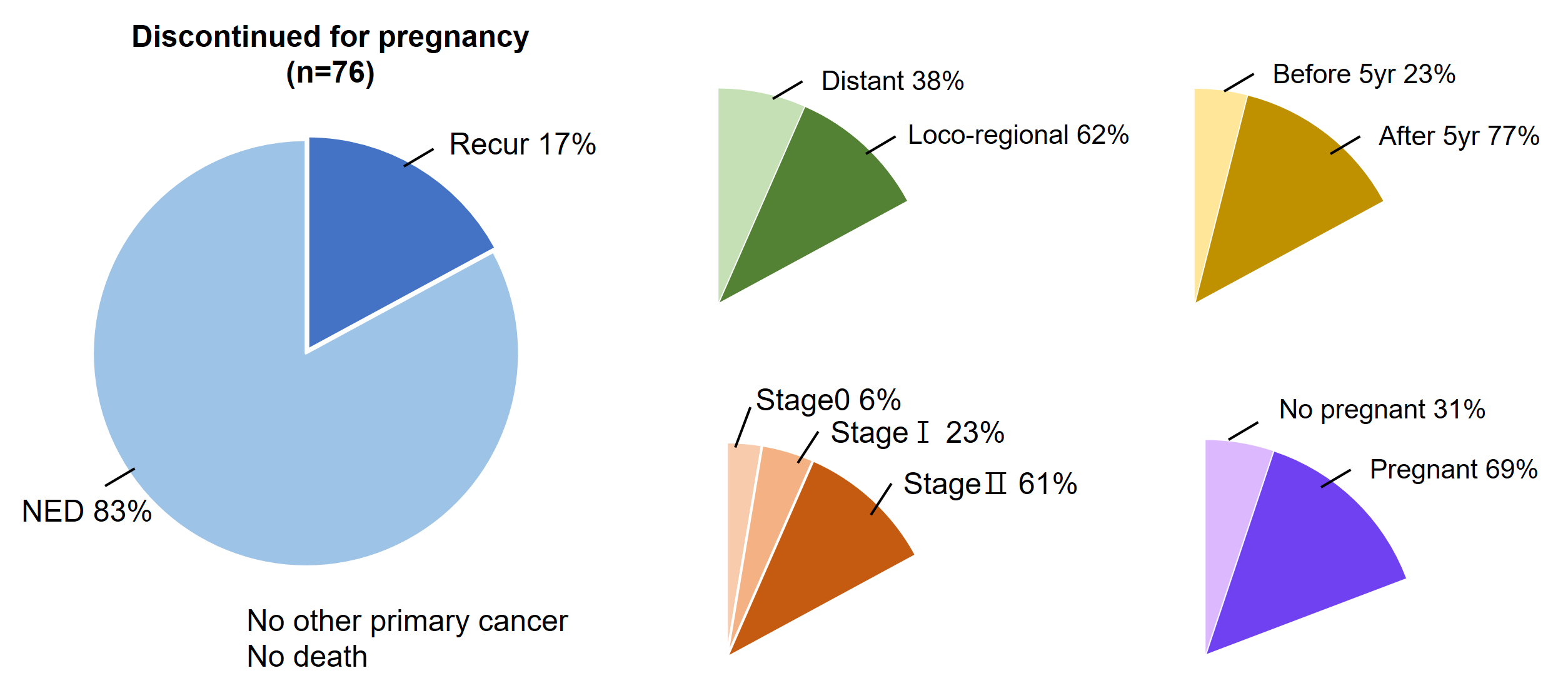


**eFigure 2. Recurrence after discontinuation of endocrine therapy**
